# Effects of Age and Sex in the Diagnosis of Type 2 Diabetes Using Glycated Haemoglobin in Japan: The Yuport Medical Checkup Centre Study

**DOI:** 10.1371/journal.pone.0040375

**Published:** 2012-07-05

**Authors:** Machiko Inoue, Kazuo Inoue, Kimihiko Akimoto

**Affiliations:** 1 Department of Community Medicine, Teikyo University School of Medicine, Tokyo, Japan; 2 Department of Community Medicine, Chiba Medical Center, Teikyo University School of Medicine, Chiba, Japan; 3 Akimoto Occupational Health Consultant Office, Tokyo, Japan; Yale School of Public Health, United States of America

## Abstract

**Background:**

We examined how the prevalence of individuals diagnosed with diabetes differs by age and sex using the diagnostic criteria of fasting plasma glucose (FPG) and/or glycated haemoglobin (HbA1c) in a large Japanese population.

**Methods:**

We conducted a cross-sectional study using a dataset of 33,959 people (16,869 men and 17,090 women) without known diabetes who underwent health checkups from 1998 to 2006. We divided the age range of the participants into six groups of similar numbers. We compared the prevalence of diabetes using the criteria of FPG ≥7.0 mmol/l (126 mg/dl), HbA1c ≥48 mmol/mol (6.5%), or both, in men and women in each age group.

**Results:**

Men had higher prevalence of diabetes than women using the criterion of either FPG or HbA1c (7.5% men vs. 3.4% women, P<0.001), or both (4.3% men vs. 1.8% women, P<0.001). HbA1c increased steadily in women through the six age groups. In the oldest group (≥66 years), the proportion of women among those diagnosed with diabetes was as high as 42.3% (215/508) using the criterion of either FPG or HbA1c, and 41.6% (116/279) using both criteria.

**Conclusions:**

Using either FPG or HbA1c, the prevalence of people diagnosed with diabetes would almost double compared to using the criterion of both scores, and this would include more elderly women than men. The impact of introducing HbA1c for diabetes diagnosis should be considered in terms of age and sex.

## Introduction

The most recent change in the diagnostic criteria for type 2 diabetes was the addition of glycated haemoglobin (HbA1c) as a criterion. In 2010, the American Diabetes Association (ADA) and an International Expert Committee proposed a threshold of HbA1c ≥48 mmol/mol (6.5%) or higher for diabetes [1]–[2]. Using the new criteria, diabetes can be diagnosed by either fasting plasma glucose (FPG), 2-h plasma glucose by oral glucose tolerance test (OGTT), and/or HbA1c.

The Japan Diabetes Society (JDS) had been working on national standardization of HbA1c measurement since the early 1990s. In 1999, it included high HbA1c as an adjunct condition when diabetes diagnosis was suspected because of a single high plasma glucose reading [3]. In 2010, the JDS proposed including elevated HbA1c as the fourth condition regarded as to indicate diabetic type in addition to elevated FPG, 2-h PG, or random plasma glucose (RPG) [4]–[5]. For the clinical use of this diagnosis, elevated PG should be confirmed by another test; however, if PG and HbA1c are measured at the same time and both are high, the diagnosis is confirmed without repeating the test [4].

The utility of HbA1c in diabetes screening is under discussion [6]–[12], even after its introduction as part of the diagnostic criteria. Advantages of using HbA1c [8] are that it is convenient because it does not require fasting, reflects long-term glycaemia, and has less day-to-day variability and greater pre-analytical stability than PG. Moreover, the measurement of HbA1c is now internationally standardized in many developed countries. However, whether people diagnosed using HbA1c have different characteristics from those diagnosed by conventional criteria remains to be investigated [11], [13]–[14], as well as how well diagnoses using HbA1c predicts future risk of end-organ complications and mortality [15]–[16]. Furthermore, reports on the effect of age [17]–[20] and sex [18] on HbA1c values raise the question of whether the use of universal cut-off values for HbA1c is appropriate [21]–[22].

Accordingly, we examined how the prevalence of diabetes diagnoses differs in age and sex using the diagnostic criteria of FPG and/or HbA1c [23]–[24] in a large Japanese population.

## Materials and Methods

We used a dataset derived from the health screening program performed by the Yuport Medical Checkup Centre in Tokyo, Japan, described in our previous studies [25]–[26]. During April 1998 to March 2006, 34,303 persons aged 15–93 years who were without known diabetes voluntarily underwent checkups. We excluded 344 because they lacked data for FPG or HbA1c, and analyzed the data of 33,959 persons (16,869 men and 17,090 women). In accordance with the Private Information Protection Law, information that might identify subjects was safeguarded by the centre. Informed consent for anonymous participation in epidemiological research was obtained at every check-up [25].

A blood sample was obtained after overnight fasting and measured at the centre's laboratory. For measurements of FPG and HbA1c levels, a Toshiba TBA-40FR Autoanalyzer (Toshiba Medical Systems, Tokyo, Japan) was used. PG level was measured using the hexokinase/glucose-6-phosphate dehydrogenase method (Denka Seiken, Niigata, Japan) with an inter-assay coefficient of variation (CV) of ≤3.0%. HbA1c level was measured by the latex immune-agglutinin method (Determiner haemoglobin A1c, Kyowa Medex, Tokyo, Japan) with an inter-assay CV of 1.7–2.1%, comparable to that of PG and aligned with JDS assigned values [25]. HbA1c values were converted to and reported as National Glycohemoglobin Standardization Program (NGSP) equivalent values (%) by adding 0.4 (%) to JDS values, using a JDS-determined formula [4].

**Figure 1:**
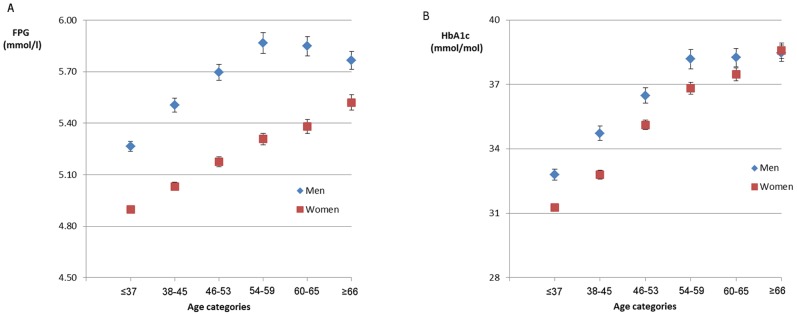
Fasting plasma glucose (FPG) (A) and glycated haemoglobin (HbA1c) (B) in six age categories. Error bars display 95% confidence intervals in each of the age categories.

We divided the age range of the participants (15–93 years, mean 51.7 with standard deviation 13.1 years) into six groups of similar size. The six age groups were ≤37, 38–45, 46–53, 54–59, 60–65, and ≥66 years. The mean values of FPG and HbA1c in men and women in each age group are in Figure 1 with error bars as 95% confidence intervals (CI). In each age group, we compared the values of men and women using a *t*-test. We described the prevalence of individuals diagnosed with diabetes using either FPG ≥7.0 mmol/l (126 mg/dl) or HbA1c ≥48 mmol/mol (6.5%), FPG alone, HbA1c alone, or both values, according to the ADA and the JDS guidelines for diabetes diagnosis [1], [4]. Prevalence rates were compared between men and women in each age category using a chi-square test. Statistical significance was determined at P<0.05. All statistical analyses were performed with PASW Statistics version 18.0 (IBM, NY, USA).

## Results

The trends of mean FPG and HbA1c values according to age categories are in Figure 1. Men's FPG values were significantly higher than women's values throughout the six age groups (P<0.001), however, the difference became smaller in age groups 60 years and over. For HbA1c, except for the ≥66-year group, men's mean values were higher than women's, and the difference also became smaller as age increased. In women, mean values for both FPG and HbA1c showed a steady increase as age increased (P<0.001). In men, no significant increase in FPG and HbA1c was observed in age groups 4 through 6 (54 years and over).

Table 1 shows the prevalence for meeting the diagnostic criteria of FPG and HbA1c, FPG only, HbA1c only, and either, by age and sex. Using either criterion, 5.4% (1841/33,959) were diagnosed with diabetes; however, among these, 56.5% (3.1% of the total population) met both criteria. Men had a consistently higher prevalence of diabetes than women using the criterion of FPG and/or HbA1c throughout the age groups (P<0.001).

**Table 1:** Comparison of prevalence of the diagnostic criteria of fasting plasma glucose (FPG) and/or glycated haemoglobin (HbA1c) by age category.

Age (years)	N	Diabetes diagnosed by							
		Both FPG and HbA1c		FPG only (HbA1c <48mmol/mol (6.5%))		HbA1c only (FPG <7.0 mmol/l)		Either FPG or HbA1c	
		Men	Women	Men	Women	Men	Women	Men	Women
≤37	6077	24 (0.8)	3 (0.1)‡	11 (0.3)	1 (0.0)†	8 (0.3 )	4 (0.1)	43 (1.3)	8 (0.3)‡
38–45	5245	63 (2.1)	8 (0.3)‡	40 (1.4)	1 (0.0)‡	10 (0.3)	6 (0.3)	113 (3.8)	15 (0.7)‡
46–53	6208	131 (4.2)	36 (1.2)‡	62 (2.0)	9 (0.3)‡	39 (1.3)	19 (0.6)*	232 (7.5)	64 (2.1)‡
54–59	5433	164 (6.8)	59 (1.9)‡	70 (2.9)	15 (0.5)‡	43 (1.8)	37 (1.2)	277 (11.5)	111 (3.7)‡
60–65	5767	191 (7.1)	82 (2.7)‡	62 (2.3)	31 (1.0)‡	57 (2.1)	47 (1.5)	310 (11.4)	160 (5.2)‡
≥66	5229	163 (6.5)	116 (4.3)‡	59 (2.4)	25 (0.9)‡	71 (2.8)	74 (2.7)	293 (11.7)	215 (7.9)‡
Total	33959	736 (4.3)	304 (1.8)‡	304 (1.8)	82 (0.5)‡	228 (1.4)	187 (1.1)*	1268 (7.5)	573 (3.4)‡

Diagnostic criteria for diabetes using FPG ≥7.0 mmol/l and/or HbA1c ≥48 mmol/mol (6.5%). Data are N (%) within age and sex categories. P-values were calculated using a chi-square test to compare men and women within the age categories. *P<0.05, †P<0.01, ‡P<0.001.

Using FPG and/or HbA1c criteria, the prevalence of diabetes increased steadily as age became higher in women, while in men, it remained similar in age groups over 54. In the oldest group (≥66 years), the proportion of women among those diagnosed with diabetes was as high as 42.3% (215/508) using the criterion of either FPG or HbA1c, and 41.6% (116/279) using both criteria.

## Discussion

Our results suggested that attention should be given to age and sex when using HbA1c in diabetes screening. As reported in previous studies [18]–[21], HbA1c and FPG values differ significantly by age and sex. The prevalence of diabetes was significantly higher in men than in women, and increased consistently in higher age groups in women. In women, HbA1c rose steadily as age increased, and in older age groups, more people were diagnosed by HbA1c than by FPG only.

Two previous studies in East Asian population reported a 2.2-fold [13] and a 2.1-fold [14] increase in the prevalence of diabetes diagnoses using either FPG or HbA1c criterion compared to using both, while our study reported a 1.8-fold increase. This might be related to higher overall prevalence of undiagnosed diabetes (5.4% by HbA1c or FPG) in our study than the others (4.1% and 3.6%) [13]–[14]. Despite these slight differences, we conclude that if HbA1c is introduced for diagnoses as proposed by the ADA, prevalence could almost double in East Asian populations as compared to the use of both FPG and HbA1c.

Our results suggested that using HbA1c would diagnose more elderly women than diagnosis using FPG alone or in combination with FPG, as in other studies [14], [27]. For the effective management of diabetes to prevent long-term vascular complications, detecting the disease at an early stage is necessary [28]. However, the impact and the effectiveness of diagnosing late onset diabetes in the elderly for the prevention of complications should also be investigated [29]. In those who are ≥66 years of age, using either HbA1c or FPG as the criterion would label one in ten elderly as having untreated diabetes. This would be approximately 3 million people in Japan, and total medical costs to treat all of them would be tremendously high. The benefit of vigorously treating mild diabetes with onset at later stage of life is still unknown compared to the early treatment of young adults who are assumed to have higher risk of developing long-term complications [29]–[30].

Our study has several limitations. First, we did not duplicate FPG measurements twice or 2-h glucose by OGTT, so we could not compare the diagnostic utility of HbA1c with these tests. For epidemiological studies, however, estimation of diabetes prevalence and incidence using a single elevated HbA1c or FPG is considered acceptable [4]–[5]. Second, the participants of our study were a healthy population who had voluntarily enrolled in a health check-up program. They might have had higher awareness in health maintenance than the general population. Screening for diabetes in a random population might reveal a higher percentage of undiagnosed diabetes by using any criteria.

Our study has a strength in the large sample size with similar numbers of men and women and the wide range of the participant' age distribution. Our results clearly illustrated the effects of age and sex on FPG and HbA1c, and showed that elderly women were more likely to be diagnosed as having diabetes by introducing HbA1c rather than using FPG alone. The need to adjust the cut-off values of HbA1c for screening of diabetes by age and sex needs to be researched considering the risk of developing long-term complications.
